# Differential regulation of neurotrophin expression in human bronchial smooth muscle cells

**DOI:** 10.1186/1465-9921-7-18

**Published:** 2006-01-29

**Authors:** Cecilia Kemi, Johan Grunewald, Anders Eklund, Caroline Olgart Höglund

**Affiliations:** 1 Department of Medicine, Division of Respiratory Medicine, Lung Research Laboratory, Karolinska Institutet and Karolinska University Hospital Solna, 171 76 Stockholm, Sweden; 2 Department of Physiology and Pharmacology, Karolinska Institutet, 171 77 Stockholm, Sweden

## Abstract

**Background:**

Human bronchial smooth muscle cells (HBSMC) may regulate airway inflammation by secreting cytokines, chemokines and growth factors. The neurotrophins, including nerve growth factor (NGF), brain-derived neurotrophic factor (BDNF) and neurotrophin-3 (NT-3), have been shown to be elevated during airway inflammation and evoke airway hyperresponsiveness. We studied if HBSMC may be a source of NGF, BDNF and NT-3, and if so, how inflammatory cytokines may influence their production.

**Methods:**

Basal and cytokine (IL-1β, IFN-γ, IL-4)-stimulated neurotrophin expression in HBSMC cultured *in vitro *was quantified. The mRNA expression was quantified by real-time RT-PCR and the protein secretion into the cell culture medium by ELISA.

**Results:**

We observed a constitutive NGF, BDNF and NT-3 expression. IL-1β stimulated a transient increase of NGF, while the increase of BDNF had a later onset and was more sustained. COX-inhibitors (indomethacin and NS-398) markedly decreased IL-1β-stimulated secretion of BDNF, but not IL-1β-stimulated NGF secretion. IFN-γ increased NGF expression, down-regulated BDNF expression and synergistically enhanced IL-1β-stimulated NGF expression. In contrast, IL-4 had no effect on basal NGF and BDNF expression, but decreased IL-1β-stimulated NGF expression. NT-3 was not altered by the tested cytokines.

**Conclusion:**

Taken together, our data indicate that, in addition to the contractile capacity, HBSMC can express NGF, BDNF and NT-3. The expression of these neurotrophins may be differently regulated by inflammatory cytokines, suggesting a dynamic interplay that might have a potential role in airway inflammation.

## Background

Allergic asthma is characterised by an inflammatory airway obstruction induced by specific allergen [1]. In addition, structural cells of the allergic airways are often hyperresponsive to non-specific stimuli, which synergises with the inflammatory response to aggravate disease [2]. While the pathogenesis of allergen-induced inflammatory airway obstruction is relatively well understood [1], we know much less about the regulation of airway hyperresponsiveness. According to current models, it involves proliferation and phenotypic changes of structural cells (e.g. smooth muscle cells, fibroblasts) and nerve cells in the airways, which contribute to enhanced airway resistance [2,3].

Neurotrophins, such as nerve growth factor (NGF), brain-derived neurotrophic factor (BDNF) and neurotrophin-3 (NT-3) were initially discovered as factors that regulate development, differentiation and survival of neurones (for review see [4]). However, neurotrophins have recently been implicated also in inflammatory responses. For example, NGF enhances survival, activation and mediator release from multiple cell types of the immune system, such as mast cells, lymphocytes and eosinophils (for review see [5,6]). In agreement with a pathogenic role in allergic asthma, elevated levels of NGF, BDNF and NT-3 have been detected in blood and locally in the airways in patients with asthma [7-10], a response that can be further augmented after allergen challenge [9,11]. In animal models, NGF and BDNF may contribute to the development of bronchial hyperresponsivness (BHR) [12-14]. A direct action on neurones may be one part of this response since NGF increases the number of neurones and the neuropeptide content in the airways [15,16]. However, NGF has also been suggested to participate in tissue remodelling processes and fibrosis in the airways [17,18], suggesting important roles for neurotrophins in many pathogenic processes that characterise pulmonary inflammation and allergic disease.

Different members of the neurotrophin family often show distinct functional effects, a phenomenon that has been mostly studied in the nervous system [19-21], but has also been observed in epithelial cells [22]. NGF and BDNF may also play distinct roles in airway inflammation. For example, antibody blockage of NGF affected early inflammatory responses in murine asthma while neutralisation of BDNF reduced only chronic airway obstruction and BHR but not inflammation [23,24]. In addition, BDNF has been shown to enhance pollen-specific IgE production while NGF and NT-3 were without effect [25]. It is unknown whether similar functional differences between the neurotrophins are operating in human airway inflammation.

The source and regulation of neurotrophin expression in the airways is not fully understood. A local NGF production by structural cells, including airway smooth muscle cells, epithelial cells, fibroblasts and infiltrating inflammatory cells has been suggested *in vivo *and *in vitro *[10,11,26-28]. Compared to NGF, less is known about the cellular sources of BDNF and NT-3 in human airways, but the presence of BDNF and NT-3 in human bronchial smooth muscle (HBSMC) and epithelium has been implied using immunohistochemistry on bronchial biopsies from non-asthmatic subjects [28]. In the present study, we used primary HBSMC to study the expression patterns of three members of the neurotrophin family, NGF, BDNF and NT-3, after stimulation with inflammatory cytokines.

Our data show that HBSMC constitutively expressed all neurotrophins analysed. NGF and BDNF, but not NT-3, were induced by IL-1β, but with markedly different kinetics. While NGF was induced more rapidly and peaked at 6 hours after the start of stimulation, BDNF was maximally induced only after 24 hours. A recent study has identified a critical role for cyclooxygenase (COX) for BDNF production [29]. This led us to explore the possibility that an intermediary mediator, such as a COX-2-derived mediator, would be involved in the IL-1β-dependent BDNF secretion. Interestingly, the induction of BDNF was shown to involve a COX-2-dependent pathway. Furthermore, the addition of Th1 and Th2 cytokines affected synthesis of NGF but affected BDNF marginally. Our data suggest that HBSMC display a differential and complex transcriptional regulation of three different members of the neurotrophin family, which may provide a framework for differential functional effects of neurotrophins in airway inflammation and asthma, controlled by HBSMC and regulated by inflammatory cytokines.

## Methods

### Culture of human bronchial smooth muscle cells (HBSMC)

HBSMC in primary culture from a healthy donor (Promocell, Heidelberg, Germany) were grown in monolayer in DMEM (Sigma-Aldrich, St Louis, MO, USA) supplemented with 10% FBS (Invitrogen, Rockville, MD, USA), 100 U/mL penicillin, 100 μg/mL streptomycin, 2 μg/mL amphotericin B (Fungizone^®^, all Sigma-Aldrich) and 0.12 IU/mL insulin (Lilly, St Cloud, France) in 25 cm^2 ^flasks (Becton Dickinson Falcon, Franklin Lakes, NJ, USA). Cells at passage 9 were used in all experiments. Cells were positive for smooth muscle specific α-actin and in light microscopy the cells displayed the reported characteristics of viable smooth muscle cells in culture [30].

### Experimental procedure

At 80% confluence cells (corresponding to 800 000 cells/25 cm^2 ^flask) were growth arrested for 24 h in a low-FBS (0.3%) insulin-free DMEM. Time-dependent pattern of neurotrophin expression was studied with and without cytokines, IL-1β 10 U/mL (Boehringer Ingelheim, Mannheim, Germany), IL-4 and IFN-γ 10 ng/mL (both R&D Systems, Oxon, United Kingdom), for 0.5, 1, 2.5, 6, 24 and 48 h in fresh low-FBS medium. Dose-dependent effects of IL-1β, IL-4 and IFN-γ were studied by stimulating cells in fresh low-FBS medium for 6, 24 and 48 h with or without increasing cytokine concentrations; IL-1β 0.1–30 U/mL, IL-4 and IFN-γ 0.1–30 ng/mL. The effects of COX-inhibition on IL-1β-dependent neurotrophin secretion were studied by using the unselective COX-inhibitor indomethacin (10 μM) and the COX-2 inhibitor NS-398 (10 μM). The chosen dose of indomethacin and NS-398 has been shown to effectively inhibit IL-1β-stimulated COX activity [31]. The inhibitors were added 1 h prior to the addition of IL-1β (10 U/mL), and NGF and BDNF were measured in the cell culture supernatants following 48 h of IL-1β stimulation.

In preliminary studies IL-1β gave a maximal increase in NGF mRNA-expression at 6 h, whereas IFN-γ gave a significant increase at 48 h. Therefore, to evaluate effects of IFN-γ on IL-1β-stimulated NGF mRNA-expression, HBSMC were treated with IFN-γ (0.1–30 ng/mL) for 42 h, where after IL-1β (10 U/mL) was added and the stimulation continued for an additional 6 h. The effects of IFN-γ on IL-1β-stimulated BDNF and NT-3 mRNA expression were studied at both 6 and 48 h of stimulation with a combination of IL-1β (10 U/ml) and IFN-γ (0.1–30 ng/ml). The effects of IL-4 (0.1–30 ng/mL) on IL-1β (10 U/mL)-stimulated NGF mRNA-expression were studied following 6 h of combined stimulation. The effects of IL-4 (0.1–30 ng/mL) on IL-1β (10 U/mL)-stimulated BDNF and NT-3 mRNA-expression were studied following both 6 and 48 h of combined stimulation.

After stimulation cell supernatants were collected, centrifuged (+4°C, 400 × g, 10 min) and stored at -70°C until analysis. Cells were collected in 1 mL RLT lysis buffer (Qiagen Inc, Valencia, CA, USA) and kept at -70°C until analysis.

### Extraction of total RNA and cDNA synthesis

Total RNA was extracted from the RLT lysis buffer using the RNeasy extraction kit and genomic DNA was removed by DNAse I (all products from Qiagen Inc), according to the manufacturer's protocol. RNA was reverse transcribed in a 20 μl final volume using 10 μl of total RNA, 20 mM random primers, 200 μM of each deoxyribonucleoside triphosphate (dNTP), 40 units RNAsin (all products from Pharmacia Biotech, Uppsala, Sweden) and 200 units SUPERSCRIPT™ II RNase H^- ^Reverse Transcriptase (Invitrogen), according to the protocol and with the buffers supplied by the manufacturer. To enable detection of eventual genomic DNA contamination, non-reversed transcribed total RNA was diluted 1:1 with water whereafter it was analysed the same way as cDNA.

### Quantification of neurotrophin cDNA by real-time PCR

The basal and IL-1β-, IL-4- and IFN-γ-stimulated NGF, BDNF and NT-3 mRNA expression was quantified following 0.5, 1, 2.5, 6, 24 and 48 h of cell culture using the ABI Prism 7700 Sequence Detection System (Applied Biosystems, Foster City, CA, USA) utilising the 5' nuclease method (TaqMan) with a two-step polymerase chain reaction (PCR) protocol (95°C for 10 min, followed by 40 cycles of 95°C for 15 sec and 60°C for 1 min). The PCR reaction was set up in a volume of 25 μl, with 2 μl cDNA diluted 1/5 and a final concentration of 1× Buffer A, 5 mM MgCl_2_, 0.05 U/μl AmpliTaq Gold (all from Applied Biosystems), 200 nM dNTP-mix (Pharmacia Biotech), 300 nM of each primer and 200 nM probe. All primers and probes were purchased from Applied Biosystems, including primers and probe for the housekeeping gene 18S rRNA, which sequences were commercially available. The neurotrophin primer and probe sequences were designed using the PRIMER EXPRESS Software (Applied Biosystems) according to the manufacturer's guidelines and the target primers and probes were for technical reasons placed within a single exon. None-reversed transcribed RNA was analysed in the same way as cDNA as a negative control. Primer and probe sequences are shown in Table 1. The probes were synthesised with the fluorescent reporter dye FAM (6-carboxy-fluorescein) attached to the 5'-end and a quencher dye TAMRA (6-carboxy-tetramethyl-rhodamine) to the 3'-end. Relative quantification of the cDNA levels were performed using the 'Relative Standard Curve method', described in detail in User Bullentin #2 (Perklin Elmer Applied Biosystems, 1997), with amplification of neurotrophins and the housekeeping gene 18S in separate tubes. Briefly, standard curves for each neurotrophin and 18S were created using five serial dilutions (1:1, 1:2, 1:10, 1:20 and 1:100) of cDNA from the human foetal fibroblast cell line HFL-1 (American Type Culture Collection, Rockville, MD, USA). By plotting the values for the threshold cycle (C_T _= the first cycle in which the amount of amplicon exceeds the threshold) against the different dilutions a standard curve was obtained. All samples were run in duplicates and the mean values were used in further analysis. The relative amount of neurotrophin mRNA in each sample was then calculated as the ratio between the neurotrophin mRNA and the housekeeping gene 18S rRNA before samples were compared.

**Table 1:** PCR primers and TaqMan probes

**NGF**	Forward	5': ACA TTA ACA ACA GTG TAT TCA AAC AGT ACT TT
	Reverse	5': CGG CAC CCG CTG TCA
	Probe	5': ACC AAG TGC CGG GAC CCA AAT CC
	Length of product (bp)	79
**BDNF**	Forward	5': AGT GCC GAA CTA CCC AGT CGT A
	Reverse	5': TAT GAA TCG CCA GCC AAT TCT
	Probe	5': TGC GGG CCC TTA CCA TGG ATA GC
	Length of product (bp)	74
**NT-3**	Forward	5': GAT AAA CAC TGG AAC TCT CAG TGC AA
	Reverse	5': GCC AGC CCA CGA GTT TAT TGT
	Probe	5': CAA ACC TAC GTC CGA GCA CTG ACT TCA GA
	Length of product (bp)	84

### Detection of neurotrophin expression by conventional PCR

Constitutive mRNA expression of neurotrophins by HBSMC was analysed following 0.5, 1, 2.5, 6, 24 and 48 h of cell culture by conventional PCR on a GeneAmp PCR System 9600 (Applied Biosystems). PCR was performed in a total volume of 20 μl using 2 μl of 1/5 diluted cDNA and a final concentration of 200 nM dNTP-mix (Pharmacia Biotech), 300 nM of each primer (Tabel 1), 1 × TaqGold-buffer II, 1.9 mM MgCl_2 _and 0.05 U/μl AmpliTaq Gold (all Applied Biosystems). The thermal cycling conditions were: 94°C for 12 min, followed by 30 cycles for 18S and 40 cycles for the neurotrophins of 94°C for 30 sec, 60°C for 30 sec and 72°C for 1 min, before ending with 72°C for 9 min. The products were analysed on a 1.5% agarose (Invitrogen) gel complemented with 0.025‰ ethidium bromide (Pharmacia Biotech), where a 1 kb DNA-ladder (Invitrogen) was run in parallel to the samples. The gels were pictured using a Kodak Digital Science 1D™ analysing system (Kodak Scientific Imaging Systems, New Haven, CT, USA).

### Quantification of neurotrophin protein by enzyme-linked immunosorbent assay (ELISA)

To quantify the NGF, BDNF and NT-3 protein levels in cell culture supernatants, commercially available ELISA kits were used, according to the manufacturer's instructions (Promega, Madison, WI, USA). All measurements were performed in duplicate. Constitutive protein secretion was analysed following 2.5, 6, 24, and 48 h of cell culture for NGF and BDNF, and 2.5, 6, 24, 48 and 72 h of cell culture for NT-3. IL-1β stimulated protein expression was analysed at 2.5, 6, 24 and 48 h for all three neurotrophins. Protein expression following IL-1β stimulation and COX-inhibition was analysed following 48 h of stimulation. Prior to analysis of NT-3 the supernatant was concentrated using the Amicon Ultra-15 Centrifugal Filter Units (Millipore, Carrigtwohill, Ireland), with a molecular weight cut off of 5,000 Da, according to the protocol obtained from the manufacturer. The detection range for NGF and BDNF was 2.0–250 pg/mL, and for NT-3 2.4–150 pg/mL.

### Statistical analysis

NGF, BDNF and NT-3 mRNA expression was formulated as the ratio of neurotrophin cDNA to 18S rRNA and expressed as neurotrophin cDNA/18S rRNA. Effects of cytokines on neurotrophins mRNA expression and protein secretion were expressed as change in mRNA (%) and protein release (pg/mL) from baseline level of time-related control. Results are presented as mean ± SEM of 3–10 independent experiments performed in duplicate. Raw data were compared using one-way analysis of variance (ANOVA) followed by Tukey's post test. Differences were considered significant at p≤0.05. All analyses were performed using GraphPad InStat 3.01 (Graph Pad Software, San Diego, CA, USA).

## Results

### Neurotrophin mRNA and protein expression in HBSMC

**Figure 1:**
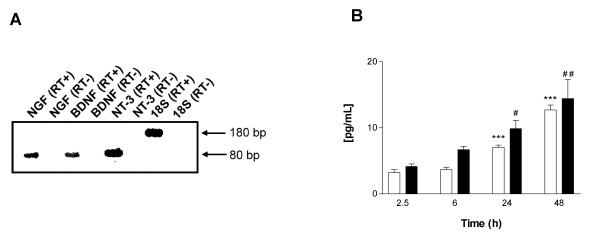
Constitutive expression of neurotrophins by non-treated HBSMC evaluated by RT-PCR and ELISA. (A) Expression of NGF, BDNF, NT-3 mRNA and 18S rRNA. Reversed transcribed (RT+) and non-reversed transcribed (RT-) mRNA or rRNA are displayed. (B) NGF (white bars) and BDNF (black bars) protein secretion determined by ELISA. Data presented as mean ± SEM of 3–4 independent experiments performed in duplicate. In (B) ***: p < 0.001 for NGF, and #: p < 0.05 and ##: p < 0.01 for BDNF, *versus *2.5 h

In order to study the expression of NGF, BDNF and NT-3 by unstimulated cultured HBSMC, we analysed cellular mRNA as well as secreted protein in the culture supernatants, at six different time points (0.5, 1, 2.5, 6, 24 and 48 h) after the start of the cultures. HBSMC constitutively expressed all three neurotrophins at the mRNA level. In Figure 1A a representative expression is pictured following 2.5 h of incubation. Furthermore, NGF and BDNF proteins accumulated in the culture supernatants over time. Hence, NGF and BDNF reached detectable levels following 2.5 h of cell culture and increased further over the next 48 h (Figure 1B). In contrast to NGF and BDNF, NT-3 protein was made in very low amounts, reaching detectable levels (3.1 ± 0.7 pg/mL) only after 72 h of culture (not shown).

### Effects of IL-1β on neurotrophin mRNA and protein expression

**Figure 2:**
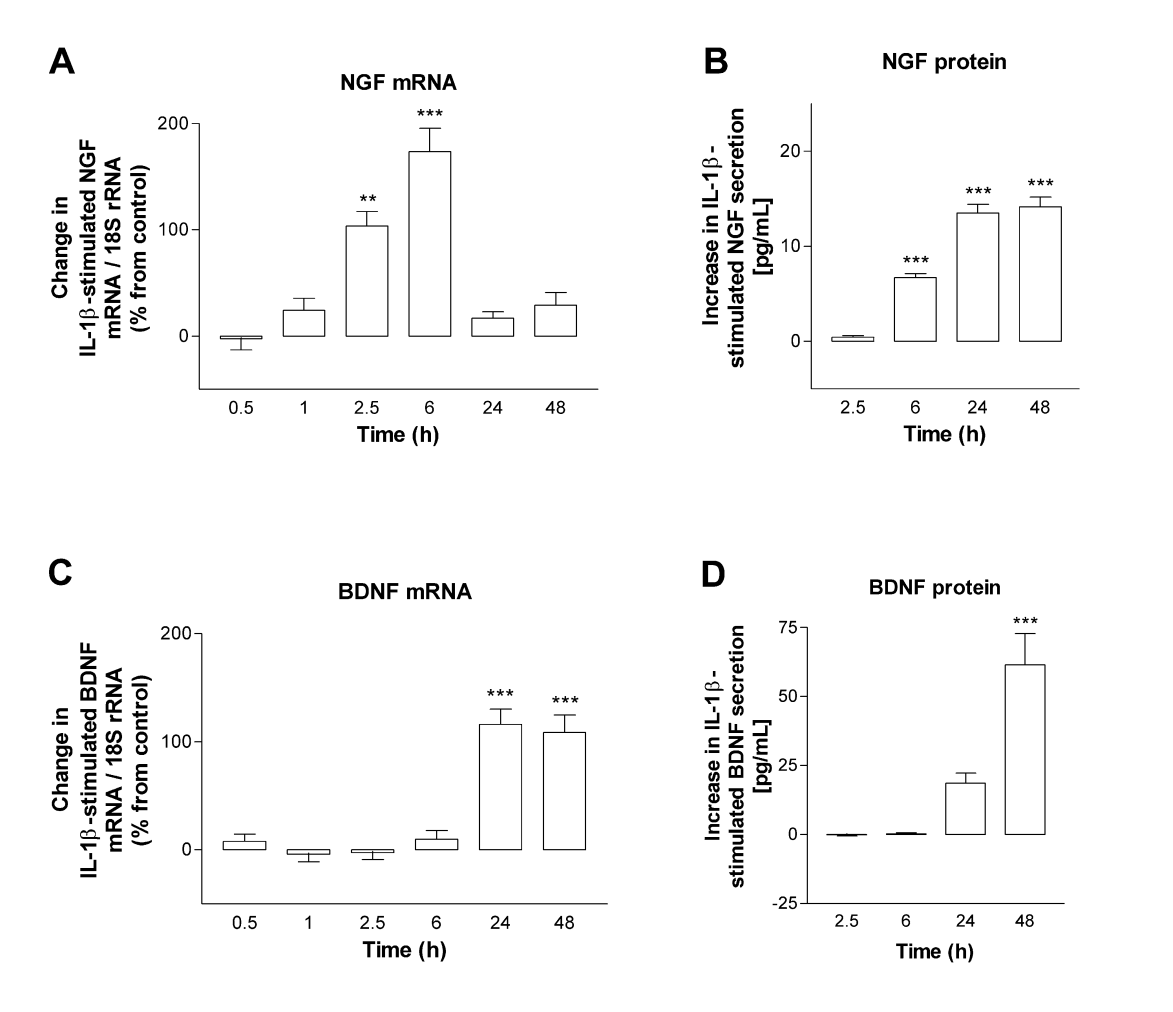
Time-course effects of IL-1β (10 U/mL) on NGF and BDNF mRNA and protein expression in HBSMC evaluated with real-time RT-PCR and ELISA, respectively. (A) NGF mRNA; (B) NGF protein; (C) BDNF mRNA and (D) BDNF protein. The IL-1β-dependent effects on neurotrophin mRNA expression and secreted protein are expressed as change in neurotrophin mRNA (%) and protein release (pg/mL) from baseline level of time-related control. Unstimulated control is set to 0 (% or pg/mL, respectively). Data are presented as mean ± SEM of 4–10 independent experiments performed in duplicate. In (A) and (C): **: p < 0.01 *versus *0.5 h, ***: p < 0.001 *versus *0.5 h. In (B) and (D): ***: p < 0.001 *versus *2.5 h.

**Figure 3:**
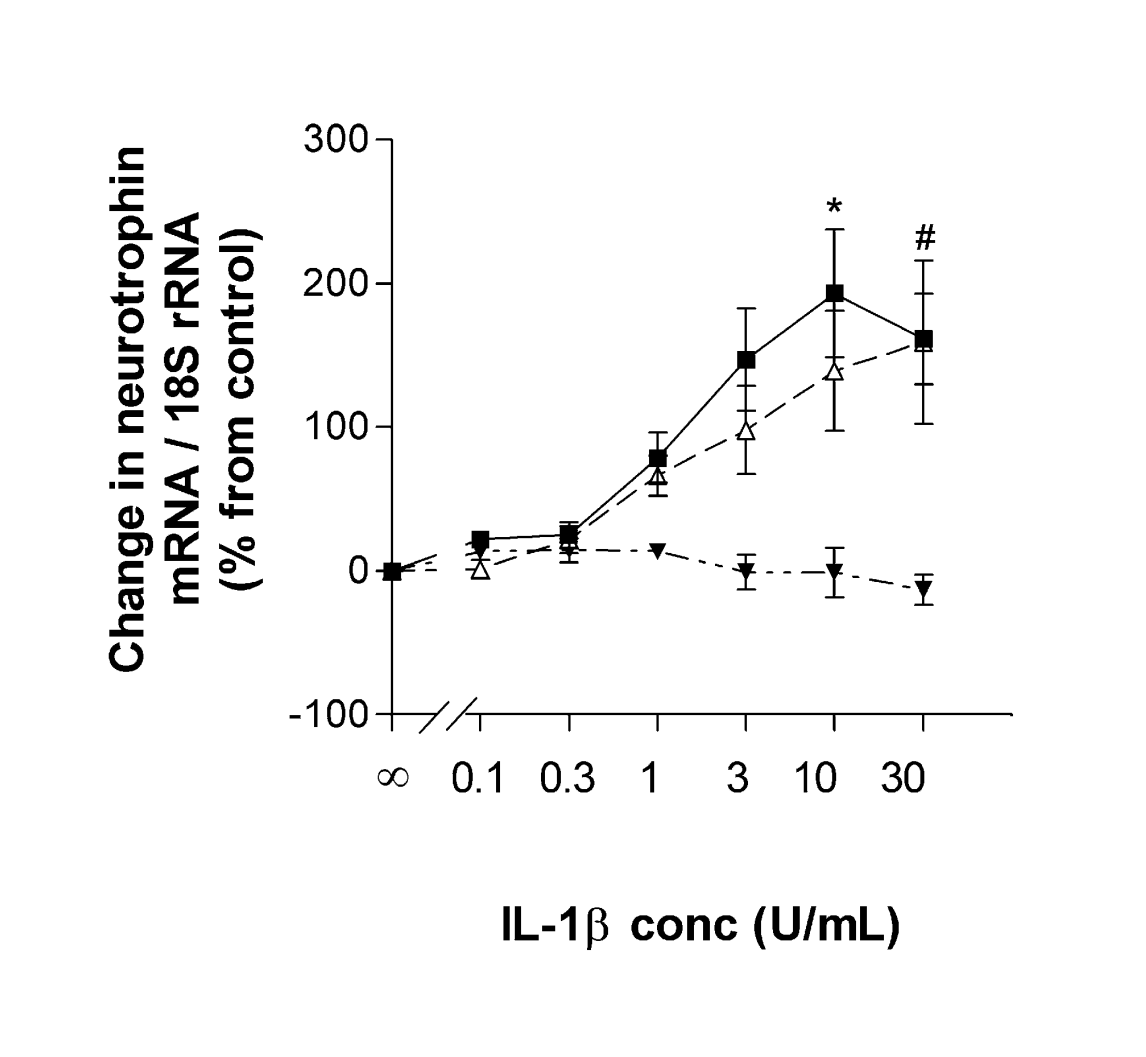
Dose-dependent effects of IL-1β on neurotrophin mRNA expression in HBSMC evaluated by real-time RT-PCR. NGF mRNA expression evaluated at 6 h (solid squares); BDNF mRNA expression evaluated at 48 h (open triangle); NT-3 mRNA evaluated at 48 h (closed triangles). The IL-1β-dependent effects on neurotrophin mRNA expression are expressed as change in neurotrophin mRNA (%) from baseline level of time-related control. Unstimulated control is set to 0 %. Data are expressed as mean ± SEM of 3 independent experiments performed in duplicate. *: p < 0.05 for NGF and #: p < 0.05 for BDNF versus unstimulated control.

We next investigated the ability of the proinflammatory cytokine IL-1β to induce neurotrophin expression in HBSMC. At 2.5 h after addition of IL-1β (10 U/mL), NGF mRNA expression was significantly increased compared to control cultures. A maximal increase was reached after 6 h of stimulation, after which mRNA levels dropped to near basal levels (Figure 2A). A corresponding increase in NGF protein secretion was detected, reaching a maximal increase after 24–48 h of IL-1β stimulation (Figure 2B). Interestingly, the induction of BDNF mRNA expression by IL-1β (10 U/mL) showed a completely different kinetics. At 6 h, no increase was seen and only at 24 h after stimulation (which was our next observation point) we observed a significant upregulation of BDNF mRNA (Figure 2C). A corresponding elevation of BDNF protein secretion was evident at 24 h, a secretion that was further enhanced at 48 h (Figure 2D). The increases in NGF and BDNF mRNA expression by IL-1β- were both dose-dependent (0.1–30 U/mL) (Figure 3). NT-3 mRNA expression was unaltered at any tested dose of IL-1β as evaluated at 2.5, 6, 24 h (not shown) and 48 h (Figure 3).

### Effects of COX-inhibitors on IL-1β-stimulated NGF and BDNF protein secretion

**Figure 4:**
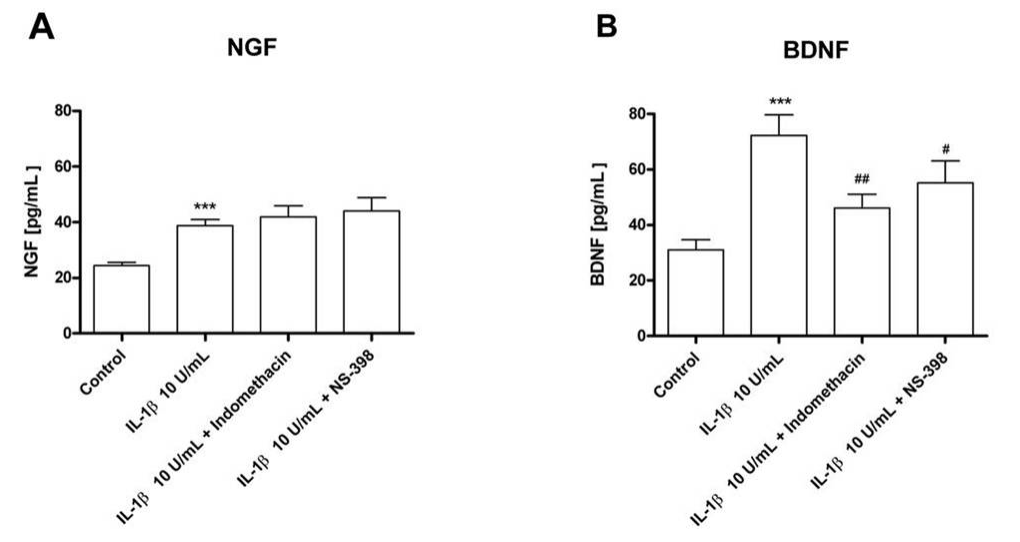
Effects of the COX-inhibitors indomethacin and NS-398 (both 10 μM) on IL-1β (10 U/mL)-stimulated NGF (A) and BDNF (B) secretion by HBSMC, evaluated by ELISA. Data are presented as mean ± SEM of 6 independent experiments. ***: p < 0.001 *versus *control, and #: p < 0.05, ##: p < 0.01 *versus *IL-1β (10 U/mL) alone.

We hypothesised that the slower induction kinetics of BDNF compared to NGF after IL-1β stimulation reflected an involvement of COX in the induction of BDNF but not of NGF. To test this, we evaluated the effects of the COX-inhibitors indomethacin and NS-398 (both 10 μM) on the secretion of these two neurotrophins. BDNF secretion after IL-1β (10 U/mL) stimulation was significantly inhibited by both indomethacin and NS-398 (Figure 4). None of the COX inhibitors altered basal NGF or BDNF secretion (not shown) and they were inefficient in preventing IL-1β-stimulated NGF secretion (Figure 4). These data suggest that the production of BDNF, but not of NGF, in HBSMC involves COX-2 and the synthesis of prostaglandins.

### Effects of IFN-γ and IL-4 on neurotrophin mRNA expression

**Figure 5:**
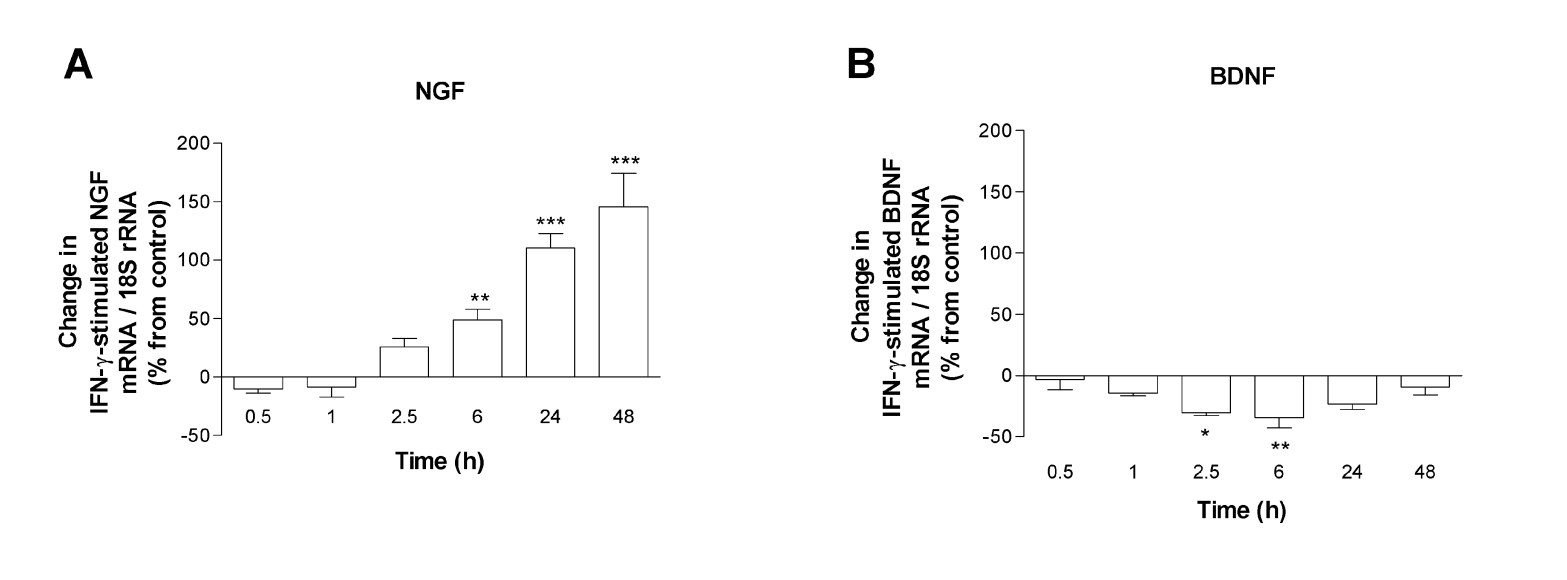
Time-course effects of IFN-γ (10 ng/mL) on NGF (A) and BDNF (B) mRNA expression in HBSMC evaluated by real-time RT-PCR. The IFN-γ-dependent effects on neurotrophin mRNA expression are expressed as change in neurotrophin mRNA (%) from baseline level of time-related control. Unstimulated control for each time-point is set to 0 %. Data are presented as mean ± SEM of 6 independent experiments performed in duplicate. *: p < 0.05, **: p < 0.01, ***: p > 0.001 *versus *0.5 h.

Allergic asthma is characterised by a disturbed balance between Th1 and Th2 cytokines towards Th2 dominance [1]. To test the hypothesis that Th1 and Th2 cytokines displayed different effects on neurotrophin gene expression by HBSMC, we treated HBSMC with either IFN-γ or IL-4 and measured the relative mRNA content of NGF and BDNF at various time points after addition of the cytokine. IFN-γ enhanced NGF mRNA expression in a time- (Figure 5A) and dose (0.1–30 ng/mL, not shown)-dependent manner, displaying a maximally enhanced NGF mRNA level at 48 h after the start of stimulation (Figure 5A). In contrast to NGF, BDNF mRNA expression was not induced. If anything, we observed a small decrease in relative BDNF mRNA content during the first 6 h of IFN-γ stimulation, after which it returned to baseline levels at 48 h after the start of the culture (Figure 5B). NT-3 expression was unaltered in response to IFN-γ under the tested conditions (not shown). In contrast to IFN-γ, IL-4 (0.1–30 U/mL) did not have any affect on basal NGF, BDNF or NT-3 expression at any of the tested intervals (0.5, 1, 2.5, 6, 24, 48 h) (not shown).

### Effects of different combinations of IL-1β, IFN-γ, IL-4 on neurotrophin mRNA expression

**Figure 6:**
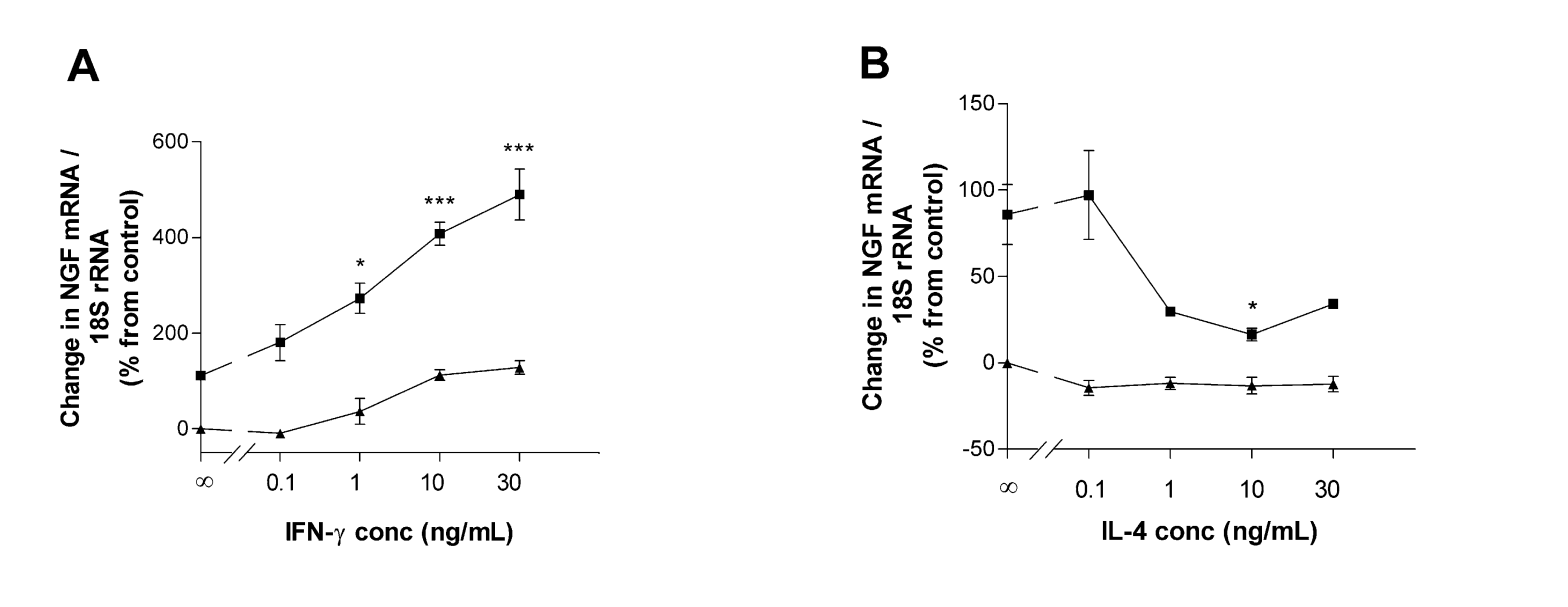
Dose-dependent effects of IFN-γ and IL-4 on IL-1β (10 U/mL)-stimulated NGF expression in HBSMC evaluated by real-time RT-PCR. Solid triangles illustrate effects of IFN-γ (A) and IL-4 (B) alone and solid squares illustrate IL-1β in combination with IFN-γ (A) and IL-4 (B). The IFN-γ- or IL-4-dependent effects on IL-1β-stimulated NGF mRNA expression are expressed as change in NGF mRNA (%) from baseline level of time-related control. Unstimulated control is set to 0 %. Data are presented as mean ± SEM of 3–4 independent experiments performed in duplicate. *: p < 0.05, **: p < 0.01, ***: p < 0.001 *versus *IL-1β (10 U/mL) alone.

During an inflammatory response *in vivo*, T cell-derived cytokines such as IFN-γ and IL-4 are likely to act in concert with proinflammatory cytokines, such as IL-1β. An interesting question was therefore whether IFN-γ or IL-4 would work in synergy with IL-1β. Since the kinetics of NGF induction in HBSMC was different between IFN-γ (peak at 48 h) and IL-1β (peak at 6 h) we cultured HBSMC with IFN-γ for 42 h and added IL-1β during the last 6 h of culture to maximise the effect of each cytokine. Under such culture conditions, we observed a potent and dose-dependent synergistic behaviour on NGF secretion by IFN-γ (0.1–30 ng/mL) and IL-1β (10 U/mL) (Figure 6A). We also tested IL-4 (0.1–30 ng/mL) in this protocol. To our surprise, we noted that IL-4 reduced the stimulatory effects of IL-1β (10 U/mL) on NGF mRNA expression, reaching a maximal reduction with an IL-4 dose of 10 ng/mL as evaluated at 6 h of combined stimulation (Figure 6B), despite the fact that IL-4 did not affect NGF secretion by itself.

In contrast, the IL-1β (10 U/mL)-stimulated expression of BDNF mRNA was not altered by IFN-γ or IL-4 at any tested dose (0.1–30 ng/mL) or time (6 and 48 h, data not shown). In addition, NT-3 mRNA expression was not altered by combining IL-1β (10 U/mL) with either INF-γ or IL-4 at any tested dose (0.1–30 ng/mL) or time (6 and 48 h, data not shown).

## Discussion

IL-1β is a pro-inflammatory cytokine of known importance in asthma pathogenesis [32]. The effect of IL-1β on NGF expression by structural cells in the airways, including HBSMC, has already been studied in some detail [27]. However, our study is the first to use HBSMC to systematically investigate how IL-1β-induced expression of NGF relates to IL-1β-induced expression of BDNF and NT-3, two other members of the neurotrophin family. This is an important question to address since these three neurotrophins have been shown to display different functional activities, including in asthmatic disease [19-22,24,25]. We found several differences in the regulation of these three neurotrophins. First, mRNA synthesis of NGF and BDNF, but not of NT-3, was stimulated in a dose-dependent fashion by IL-1β. Since nonstimulated HBSMC expressed mRNA from NT-3, the lack of induction of NT-3 cannot be explained by lack of basal gene transcription. Rather, differences in critical IL-1β response elements in the NT-3 gene may be crucial. Alternatively, NT-3 expression may require a different set of intermediate factors compared to NGF and BDNF that may be lacking in our HBSMC. It is interesting to note that NT-3 expression, but not NGF or BDNF expression, is transcriptionally downregulated in chronic obstructive pulmonary disease, suggesting that cells from the airways may be poor at producing NT-3 compared to other neurotrophins [33].

A second difference we observed was that the two neurotrophins that were induced by IL-1β (NGF and BDNF) showed markedly different kinetics of induction. While NGF mRNA induction was transient and high already at 6 h after addition of IL-1β, BDNF showed a more sustained expression pattern with a slower onset and a maximal induction after 24 h. With regard to the kinetics of NGF induction, our data is in line with that of Freund and co-workers, who similarly demonstrated a rapid and transient increase of NGF by IL-1β in airway smooth muscle cells, although Freund and co-workers reported a maximal upregulation at 2.5 h of IL-1β stimulation [27]. It may be speculated that the difference in time-course effects by IL-1β on NGF expression is dependent on the origin of the smooth muscle cells. Hence, the question of whether cells from different locations in the bronchial tree have different synthetic properties needs further investigation. In both our, and in the study by Freund and co-workers, NGF protein induction was detected with a significant delay. The dramatic differences in mRNA and protein induction between NGF and BDNF shown in our study demonstrate remarkable heterogeneity in the regulation of neurotrophin expression in HBSMC. One possible explanation for this difference could be the involvement of additional intermediary mediators in BDNF induction. Our finding that secretion of BDNF, but not NGF, was inhibited by the unselective COX inhibitor indomethacin and the COX-2 inhibitor NS-398 imply the prostaglandins as such intermediary mediators. A regulatory role of COX-2 in BDNF secretion adds to recent data showing that prostaglandins can stimulate the expression of BDNF in mouse astrocytes [34] and that COX inhibitors are able to counteract BDNF-mediated effects of spatial learning in a mouse in vivo model [29]. Interestingly, Toyomoto and co-workers found that also NGF expression was stimulated by prostaglandins [34]. More work is needed to investigate whether our observation of a differential role of COX inhibitors on NGF and BDNF secretion in HBSMC will also be observed *in vivo*.

We also found that the lymphocyte-derived cytokines IFN-γ and IL-4 affected neurotrophin expression differently. For example, the Th1 cytokine IFN-γ induced NGF expression but not BDNF expression, and IFN-γ synergised with IL-1β to enhance NGF gene transcription, implying an important role for IFN-γ in the airway inflammation. In addition, we found IL-4, a typical Th2 cytokine, to downregulate IL-1β stimulated NGF expression. In relation to the asthma, these results are surprising, but may reflect a more complex involvement of Th1/Th2 cytokines in the asthmatic airway inflammation, as also suggested from recent studies in humans [35,36] as well as from animal models of asthma [37]. More work is needed to clarify the role and pathogenic importance of Th1/Th2 cytokines and neurotrophin release in asthma pathogenesis.

Proliferation and survival of mast cells, eosinophils and lymphocytes in the airways may be of importance in establishment and maintenance of a chronic inflammation. In asthmatic subjects, NGF, BDNF, NT-3 and NT-4 significantly enhanced airway eosinophil survival [38]. Mast cells located in human asthmatic bronchus have been demonstrated to express the neurotrophin receptor TrkA [11] and NGF may enhance mast cell proliferation and survival [6]. Since mast cells have been shown to be in close contact with smooth muscle cells in the asthmatic bronchus [39], smooth muscle cell-derived NGF has the potential to influence airway mast cells. B and T lymphocytes, including CD4^+ ^T cells, express the neurotrophin receptors TrkA and TrkB, and NGF has been shown to enhance proliferation of B and T cells and survival of B cells [6]. Thus, there is evidence for a broad regulatory role for neurotrophins in the airways. In addition to cells of the immune system, neurones may also be targets for the neurotrophins in the airways. Hence, NGF has been shown to increase the number of neurones and the neuropeptide content in the airways [15,16], and both NGF and BDNF may evoke airway hyperreactivity in the airways [12-14]. In this respect, it is interesting to note that while both NGF and BDNF have been shown to evoke airway hyperreactivity in animal studies [12-14], they may have distinct roles in allergen-dependent airway obstruction [24]. Hence, NGF may play a role in the early airway response (EAR) in asthma, since anti-NGF attenuated the early allergen-induced bronchoconstriction [13,40,41], and NGF causes histamine release from mast cells [42] and basophils [43]. Anti-NGF-treated mice also reduced the eosinophil recruitment to the airways and decreased IL-4 and IL-5 production [40], and IL-1β-induced airway hyperactivity in isolated human bronchus [44]. BDNF, on the other hand, seems not to influence the EAR but rather to abrogate chronic airway obstruction [23]. Thus, NGF and BDNF may influence different phases of the allergic response. Interestingly, there are also indications of a differential *in vivo *production of the neurotrophins in allergic airway inflammation. Hence, following allergen challenge, NGF levels in bronchoalveolar lavage were increased fourfold at 18–24 h, whereas the increase in BDNF peaked after 1 week [24]. An interesting possibility is that that the differential functional effects and secretion observed between NGF and BDNF *in vivo *might somehow reflect a differential release patterns, such as the one described in our study.

In the present study, HBSMC were obtained from a healthy donor. An important next step will be to obtain airway smooth muscle cells from asthmatic donors, and ask whether such cells are similar to HBSMC from normal individuals or in some way biased towards production of a different set of neurotrophins [45]. Also, an important next step will be to test whether inflammatory and asthma-associated mediators might have different effects on asthmatic as compared to non-asthmatic bronchial smooth muscle [45,46].

## Conclusion

In conclusion, this study shows that HBSMC are a source of NGF, BDNF and NT-3 in the airways, that IL-1β, IFN-γ and IL-4 may alter this production differently, and that the IL-1β-dependent BDNF, but not IL-1β-dependent NGF, secretion is COX-2-dependent. Taken together, we propose a paracrine interaction between smooth muscle cells, neurones, inflammatory cells and structural cells in the vicinity, in which neurotrophins derived from bronchial smooth muscle may promote airway hyperreactivity, inflammation and tissue remodelling.

## Competing interests

The author(s) declare that they have no competing interests.

## Authors' contributions

CK carried out the majority of the experiments, did the statistical analysis and participated in writing the manuscript. JG participated in the design of the study and writing the manuscript. AE participated in the design of the study. COH conceived of the study and its design, performed its co-ordination, did parts of the experiments and statistical analysis and participated in writing the manuscript. All authors have read and approved the final manuscript.
